# Supplementary material for: Epidemiology of kerosene poisoning in Saudi Arabia: a retrospective analysis
Source: PeerJ. 2025 Mar 19;13:e19094. doi: 10.7717/peerj.19094 (PMC11929502; doi:10.7717/peerj.19094)
Supplement: Supplemental Information 1 [file peerj-13-19094-s001.pdf]

Region…………………………………………… **Name of Hospital/Center**…………………………………
Date of reporting (/ / G.)

Patient information

Name (……………………………………………………..) **Patient's ID / IQAMA/ Passport** ………………………….
Tel. / Mobile No.(……………………..……)
Nationality: ☐Saudi ☐ Other, Specify (………………………………………….)
Age: ☐(<1 year) ☐(1-5y) ☐(6-12y) ☐(13-19y) ☐(20-39y) ☐40 years and above **Gender** : ☐Male ☐Female
Weight (Kg) : ……………..
Place of incidence: ☐ Home ☐School ☐ Farm ☐ Workplace ☐Other, specify (…………………………….)
Date &Time of exposure: Date (/ /) Time (……..) PM ☐AM ☐

2. This part if to be filled by **Doctor**

☐**Condition of Patient at time of arrival to Hospital**:- ☐ Stable ☐Deteriorated ☐ **DEATH** *

Type of Poisoning

☐**Drug Over dosage:** Name of Drug(………………………………………………………………………………………….)
Name of Group:
1.☐Analgesic & Antipyretics 2.☐Antibiotics 3.☐Antihistaminic 4.☐Antihypertensive 5.☐Antidiabetics
6.☐Anti asthmatic 7.☐Antiemetic 8.☐Antiepileptic 9.☐Antipsychotic 10.☐Contraceptive 11☐Herbl Drug
12.☐Vitamins 13.☐Iron preparations 14.☐Others(………………………………………………)15.☐Unknown

☐**Chemical Poisoning:** Name of Substance(………………………………………………………………………………….)
Main Use:- 1.☐Insecticide 2.☐Rodenticide 3.☐Fungicide 4.☐Herbicide 5.☐Veterinary 6.☐Antiseptic 7.☐Disinfectant
8.☐Cleansing substance 9.☐Fuel 10.☐Carbon Monoxide 11.☐*Methanol (Methyl Alcohol) 12.☐*Aluminium Phosphide
13.☐Others(………………………………………………………)14.☐Unknown

Physical form of Poisoning Substance: ☐Solid ☐Powder ☐Liquid ☐Gas ☐ Other, specify ………………..
Circumstances of Exposure ☐ Unintentional ☐Intentional ☐Occupational ☐Unknown
Route of Exposure : ☐ Oral ☐ Inhalation ☐ Dermal ☐Injection ☐Others(………………………………)
Date/ Time of appearance of Signs & Symptoms: Date (/ /) Time: (………………) PM☐ AM ☐
Sign and Symptoms: ☐Nausea ☐ Vomiting ☐ Abdominal pain ☐ Diarrhea ☐ Headache ☐ Fever ☐ Weakness
☐ Skin rash ☐ Difficulty in breathing ☐ Blurred vision ☐ Constricted pupil ☐ Dizziness ☐Disorientation ☐ Seizure
☐ Loss of consciousness ☐ Coma ☐Other Symptoms:………………………… ……………………………………………

Laboratory Investigation Requested

Hospital Laboratory<br>Sample No. ………………………………………	Samples sent to Toxicological Center<br>Sample No. ……………………………………
Blood Sample ☐Yes ☐NO	Blood Sample ☐Yes ☐NO
Urine Sample ☐Yes ☐NO	Urine Sample ☐Yes ☐NO
Gastric Lavage ☐Yes ☐NO	Gastric Lavage ☐Yes ☐NO
	Environmental Sample of Poisoning Substance ☐Yes ☐NO

Antidote used:☐Activated charcoal ☐N-Acetylcystine ☐Atropione ☐Cyanide antidote kits ☐Deferoxamine
☐Fomepizole ☐Naloxone ☐Pralidoxime ☐Pyridoxine hydrochloride B6 ☐Dimercaprol (BAL)
☐Other (specify the name)…………………………………………………

Management : ☐No Admission in Hospital ☐Admission in Hospital ☐ DAMA
Outcome: ☐Recovery ☐ *Death
